# Community-based maternal and child health project on 4^+^ antenatal care in the Democratic Republic of Congo: a difference-in-differences analysis

**DOI:** 10.1186/s12978-019-0819-1

**Published:** 2019-11-01

**Authors:** Hocheol Lee, Sung Jong Park, Grace O. Ndombi, Eun Woo Nam

**Affiliations:** 1 Department of Health Administration, Graduate School, Yonsei University, 1 Yonseidae-gil, Wonju, 220-070 Republic of Korea; 2 Yonsei Global Health Center, Yonsei University, Wonju, Republic of Korea; 3 Department of Applied Statistics, Graduate School, Yonsei University, 1 Yonseidae-gil, Wonju, 220-070 Republic of Korea

**Keywords:** 4^+^ ANC services, Difference-in-differences analysis, KOICA, Yonsei Global Health Center, The Democratic Republic of Congo

## Abstract

**Background:**

Despite efforts to achieve the Millennium Development Goals, the maternal mortality ratio in the Democratic Republic of Congo was 693 per 100,000 in 2015—the 6th highest in the world and higher than the average (547 per 100,000) in sub-Saharan Africa. Antenatal care (ANC) service is a cost-effective intervention for reducing the maternal mortality ratio in low-income countries. This study aimed to identify the intervention effect of the maternal and child health care (MCH) project on the use of four or more (4^+^) ANC services.

**Methods:**

The MCH project was implemented using the three delays model in Kenge city by the Ministry of Public Health (MoPH) of the DRC with technical assistance from Korea International Cooperation Agency (KOICA) and the Yonsei Global Health Center from 2014 to 2017. Furthermore, Boko city was selected as the control group. A baseline and an endline survey were conducted in order to evaluate the effectiveness of this project. We interviewed 602 and 719 participants in Kenge, and 150 and 614 participants in Boko in the baseline and endline surveys, respectively. We interviewed married reproductive-aged women (19–45 years old) in both cities annually. The study instruments were developed based on the UNICEF Multiple Indicator Cluster Surveys. This study used the homogeneity test and the binary logistic regression difference-in-differences method of analysis.

**Results:**

The odds of reproductive-aged women’s 4+ ANC service utilization at the intervention site increased 2.280 times from the baseline (OR: 2.280, 95% CI: 1.332–3.902, *p* = .003) as compared to the control site.

**Conclusions:**

This study showed that the KOICA MCH project effectively increased the 4^+^ ANC utilization by reproductive-aged women in Kenge. As the 4+ ANC services are expected to reduce maternal deaths, this project might have contributed to reducing maternal mortality in Kenge. In the future, we expect these findings to inform MCH policies of the MoPH in the DRC.

## Plain English summary

The maternal mortality ratio (MMR) in the Democratic Republic of Congo is the sixth-highest in the world. In previous studies, the MMR in low-income countries was reduced by providing antenatal care (ANC) services. The World Health Organization also recommends that pregnant women should receive ANC services more than four times (4^+^) before giving birth. This study examined the impact of the maternal and child health (MCH) intervention using a difference-in-differences analysis. In this study, MCH interventions were provided to reproductive-aged women living in Kenge city from 2014 to 2017. Boko city was selected as the control city for comparative analysis. The MCH intervention activities included educational programs for reproductive-aged women, awareness-building programs using radio broadcasts, ambulance provision, a training program for health workers, construction of a health facility, and provision of equipment at the health facility. Reproductive-aged women participated in interviews: 602 and 719 women in Kenge; and 150 and 614 women in Boko in the baseline and endline surveys, respectively. The results of this study showed that the 4^+^ ANC service utilization rate was 2.28 times higher at the end of the intervention in 2017 as compared to before intervention in 2014. As the 4+ ANC service utilization has shown to have contributed to reduce the MMR, this study might inform the MoPH MCH policies in the DRC.

## Background

The United Nations (UN) adopted the Millennium Development Goals (MDGs) in September 2000 to increase the mother’s health status and decrease the maternal mortality ratio (MMR) worldwide. Although the global MMR per 100,000 newborns (MDG 5) decreased by 64% from 330 in 2000 to 210 in 2013, it has not been enough to achieve MDG 5 [1]. Therefore, UN member countries announced a sustainable development goal (SDG) target to decrease the MMR per 100,000 live births to 70 by 2030 [2]..

In sub-Saharan Africa, the MMR decreased by 49% between 2010 and 2013, but it was still significantly higher than the worldwide average [3]. Despite efforts to achieve the MDGs and SDGs, the MMR in the Democratic Republic of Congo (DRC) was 693 per 100,000 in 2015—the 6th highest in the world and higher than the average (547 per 100,000) in sub-Saharan Africa [4, 5]. Obstetrical complications such as bleeding, eclampsia, sepsis, and unsafe abortions accounted for nearly 80% of the cases of MMR, and the remaining 20% were caused by underlying diseases [6]. Primary health care that includes maternal and child health (MCH) services are essential for decreasing the MMR [7]. According to the results of studies from 2016, the MMR can be reduced by regular visits to health facilities that provide antenatal care (ANC) [8].

Previous studies showed that ANC is a cost-effective way of reducing the MMR in low-income countries [9]. The World Health Organization (WHO) recommends at least four visits (4^+^) to ANC services during pregnancy. ANC typically includes an obstetrical examination to determine complications, a tetanus toxoid immunization, intermittent preventive treatments for malaria during pregnancy, and the identification and management of infections including HIV, syphilis, and other sexually transmitted infections [5]. Moreover, the overall MCH can be improved by receiving 4^+^ ANC visits at health facilities [10].

The Ministry of Public Health (MoPH) in the DRC also identified ANC as a beneficial health service for improving MCH and recommended that pregnant women receive 4^+^ visits to ANC services [11]. Despite the recommendations by WHO and MoPH in the DRC, only 48% of pregnant women received ANC services more than four times [5]. Meanwhile, 85% of women received only 1^+^ ANC services: 92% in urban areas and 80.9% in rural areas [11]. This means that while pregnant women in the DRC visited health facilities for ANC services, the number of women that made regular visits was low.

According to previous studies in the DRC, the most effective way of providing sustainable ANC services is by attracting participants through community health workers, also known as *relais communautaires* (RECO) [12]. Studies have shown that the impact of RECO activity is expected to be a significant factor for the utilization of ANC services by pregnant women in the DRC [8]. Despite their positive impact on health outcomes, the DRC faces a RECO capacity shortage; their health worker numbers are not proportional to the population percentage [12, 13].

According to Thaddeus and Maine [14], an MCH intervention must focus on three steps to effectively reduce the MMR, which they refer to as the three delays model. According to this model, these delays are: 1) delays in decision-making to seek care; 2) delays in arrivals to health facilities; and 3) delays in the provision of adequate care. These three delays can be reduced to improve the ANC utilization by developing appropriate interventions [14]. Therefore, KOICA, in cooperation with MoPH of the DRC under the official development assistance (ODA) projects for low income countries, designed and implemented an MCH intervention project based on the three delays model. KOICA funded Yonsei Global Health Center (YGHC) to implement and evaluate this MCH project. YGHC, in a previous study, calculated the cost-benefit ratio (BCR) of this MCH project at 3.41, which means that the project was “more than 3 times more likely to provide benefits than cost” [15]. Although this project calculated the BCR, the impact of the MCH intervention project on the ANC service utilization by the reproductive-aged women in the DRC remains unknown. Therefore, this study aimed to measure the effect of the KOICA MCH project on the 4+ ANC service utilization rate among the reproductive-aged women in the DRC using the three delays model.

## Methods

### Study design

**Fig. 1:**
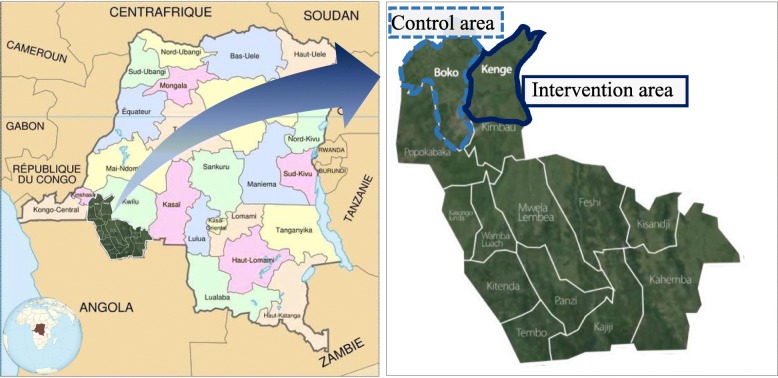
Map of intervention and control site – Kenge and Boko cities – in Kwango district

This study used a community-based, quasi-experimental, pre- and post-, comparison group study design in the DRC. KOICA MCH intervention project based on the three delays model was implemented for 3 years and 7 months from February 2014 to July 2017 in the DRC. The project was conducted in Kenge city in the Kwango district, roughly 120 km from the DRC capital, Kinshasa. The target group was 15–49-year-old reproductive-aged women, who constituted 18.7% of the total population of Kenge (50,281). The project team evaluated the effect that the MCH project had on the target group each year. The baseline survey was conducted in October 2014, and the endline survey in May 2017. Boko city, an hour away from Kenge, was the control group. The Ministry of Health recommended this city because no MCH project was being implemented at this site. In addition, both Boko and Kenge cities belonged to the same administrative zone in the Kwango district, and they had similar population sizes and comparable distributions of religion and ethnic groups (Fig. 1).

### Intervention activities

**Fig. 2:**
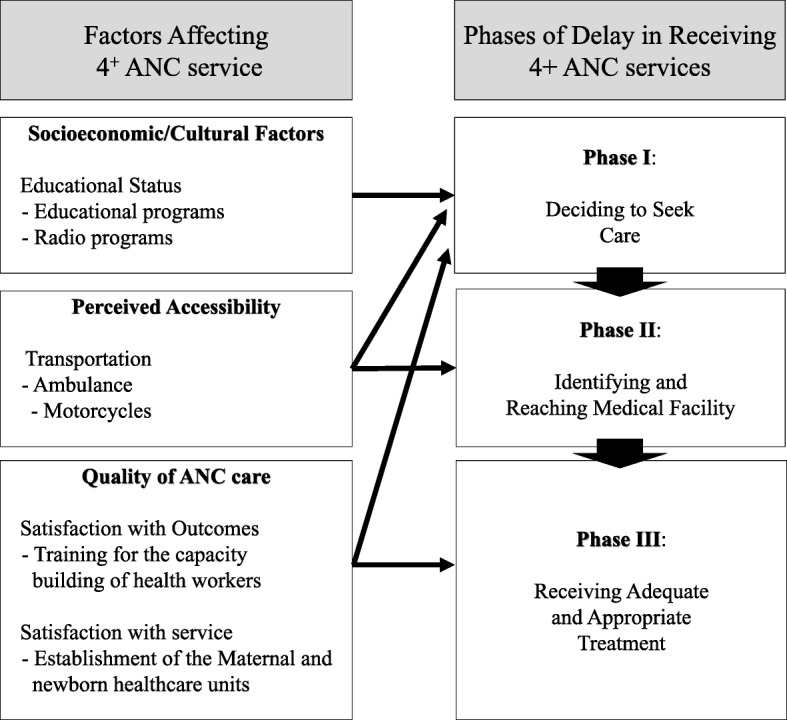
Intervention activities in the three delays model

The KOICA MCH project was developed by the YGHC based on the three delays model (Fig. 2) [14].

The first phase of the project included educational programs to increase the awareness of and promote MCH services among community residents to address the first delay of seeking ANC from health facilities. Educational programs were provided by RECO visiting each household once a month in cooperation with national health programs such as the Programme National de Santé de la Reproduction (PNSR), Programme National d’Approvisionnement en Médicaments Essentiels (PNAM), Programme National de Lutte contre les Infections Respiratoires Aigües (PNIRA), Programme National de Nutrition (PRONANUT), Programme National de Lutte contre les Maladies Diarrhéiques (PNLMD). In addition, promotional activities for reproductive-aged women via radio broadcasting and signboards were used to increase program awareness. The radio broadcasts were sent out in 30-s intervals four times a day at 7:20 AM, 8:50 AM, 6:00 PM, and 7:30 PM in the local languages of Lingala and Kikongo. The radio programs covered seven themes, including “introducing the MCH project,” “promotion of awareness of disease control for children under-five,” “health facility delivery,” “guidance for using emergency transportation,” and “continuously providing MCH intervention.” Signboards were installed to indicate the locations of secondary hospitals, health centers, and health posts.

The second phase of the program addressed the second delay of reaching the health facility to receive ANC by pregnant women during the pregnancy. Therefore, the KOICA provided a secondary hospital with an ambulance (a Toyota Land Cruiser). They also provided motorcycles (a Yamaha AG100) to 23 health centers per each health zone—i.e. “Aires de Santé (AS)”— as emergency transportation for referrals to health facilities.

Finally, the third phase of the program aimed to ensure that patients received adequate and appropriate treatment focused on ANC at health facilities. Therefore, the project team provided information regarding the capacity building of health workers to improve the quality of health services, the establishment and operation of maternal and newborn healthcare units (MNUs), and the provision of essential medicines. Training for the capacity-building of 76 health workers such as chief nurses, nurses, midwives, pharmacists, and nutritionists was conducted ten times in cooperation with PNSR, PNIRA, PNAM, and PRONANUT. The MNUs were a combination of obstetrics and gynecology outpatient clinics, delivery rooms, pediatric wards, and education rooms. The average number of deliveries per month in the MNUs was 191.

### Sampling

**Fig. 3:**
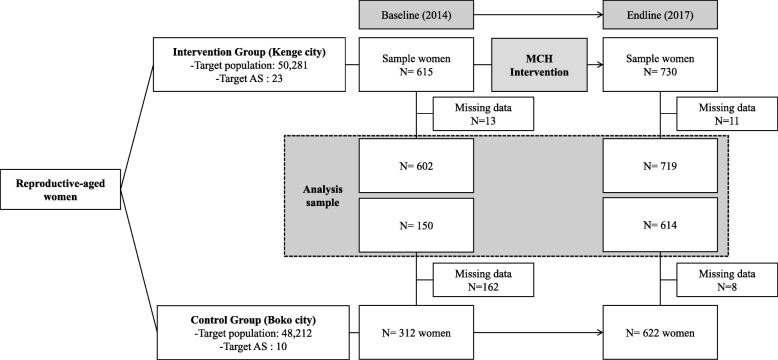
Flow diagram of the study

The sample size was calculated using the Raosoft calculator using a 95% confidence interval and a 5% margin of error. The sample population was 9403, which is 18.7% of Kenge’s total population (50,281) [16]. Therefore, the minimum sample size was determined to be 382 participants. Reproductive-aged women were selected through the Probability Proportionate to Size (PPS) method in 23 Aires de Santé (AS; health zones) in Kenge and ten AS in Boko. We randomly selected a minimum of 30 households per AS in Kenge and Boko based on the Central Limit Theorem [17]. Additionally, we selected one reproductive-aged woman from each household. This sampling method ensured that the sample was representative of the populations from each AS. Therefore, these participants were community-based cross-sectional samples for each year (Fig. 3). In the baseline, we collected data from 602 women in Kenge and 150 women in Boko. In the endline, we collected data from 719 women in Kenge and 614 in Boko. We checked the data quality to ensure it was appropriate for analysis. Consequently, we excluded cases or records with missing and censored data. The final sample consisted of 615 women in Kenge and 312 women in Boko in the baseline, and 719 women in Kenge and 614 women in Boko in the endline.

### Data collection

Face-to-face interviews were conducted by visiting households with reproductive-aged women. The trained female interviewer questioned the selected reproductive-aged women, and the answers were noted in the questionnaire. We obtained written informed consent from all the respondents (i.e., reproductive-aged women/respondents on behalf of children under 5 years). Special emphasis on the respondents’ rights to refuse to answer questions was provided to the participants. Each survey team consisted of a team manager, a supervisor, and two interviewers. The manager directed the team and took care of the logistics while the supervisor ensured the quality of data. Each questionnaire took about 1 h to complete. Thus, out of 2279 women respondents, data of 2085 women (91.4%) were analyzed.

### Study instrument

The questionnaire used in this study was adapted from the UNICEF Multiple Indicator Cluster Surveys 2011 [18]. After three meetings with three health experts from the School of Public Health at Kinshasa University in the DRC, this questionnaire was psychometrically tested to ensure the validity and reliability of the data. Thereafter, the questionnaire was validated by conducting a pre-survey among 36 reproductive-aged women in Maluku, which is similar to Kenge.

### Statistical analysis

An evaluation of the MCH project effects in Kenge was conducted using IBM SPSS Statistics 24.0, and the detailed methods are as follows:

#### Homogeneity test

This study conducted the homogeneity test to confirm that Kenge and Boko were identical before the intervention. In this study, the homogeneity, which included the dependent variable (4^+^ ANC utilization rate), used six of the WHO recommended indicators to evaluate the MCH intervention [19]. These six indicators were ANC, PNC, neonatal examination, skilled birth attendants, exclusive breastfeeding, and using an improved water source. Table 1 shows the results of the chi-squared test of homogeneity between Kenge and Boko from the baseline to endline survey. In the case of the dependent variable in the baseline survey data, the 4+ ANC utilization rates were significantly different between Kenge and Boko at *p* = .033, while, In the endline (2017), these two cities were significantly different at *p* < .000. Using an improved water source showed a significant difference between Kenge and Boko from baseline to endline.

**Table 1:** Homogeneity test of the major achievements among the WHO’s 11 MCH indicators from 2014 to 2017

	Baseline (2014)			Endline (2017)		
	Group		χ^2^(*P*)	Group		χ^2^ (*P*)
	Intervention	Control		Intervention	Control	
Antenatal care						
≥ 4	229 (41.4%)	41 (31.3%)	4.533 (.033)	370 (53.4%)	251 (43.4%)	12.727 (.000)
≤ 3	324 (58.5%)	90 (68.7%)		323 (46.6%)	328 (56.6%)	
Postnatal care						
Yes	290 (57.1%)	55 (46.6%)	4.280 (.039)	594 (91.7%)	436 (80.7%)	30.494 (.000)
No	220 (42.9%)	63 (53.4%)		54 (8.3%)	104 (19.3%)	
Neonatal examination						
Yes	218 (47.8%)	27 (48.2%)	0.003 (.954)	651 (90.8%)	543 (88.9%)	1.346 (.246)
No	238 (52.2%)	29 (51.8%)		66 (9.2%)	68 (11.1%)	
Skilled birth attendants						
Yes	507 (92.0%)	117 (89.3%)	0.993 (.319)	641 (89.5%)	543 (88.4%)	0.401 (.527)
No	44 (8.0%)	14 (10.7%)		75 (10.5%)	71 (11.6%)	
Exclusive breastfeeding						
Yes	254 (53.0%)	103 (68.6%)	.889 (.509)	482 (67.9%)	267 (43.7%)	78.260 (.000)
No	225 (47.0%)	47 (31.4%)		228 (32.1%)	344 (56.3%)	
Using an improved water source						
Yes	101 (18.3%)	6 (4.6%)	14.841(.000)	232 (32.4%)	118 (19.3%)	29.100 (.000)
No	452 (81.7%)	124 (95.4%)		485 (67.6%)	494 (80.7%)	

#### Binary logistic difference-in-differences analysis

We conducted a binary logistic difference-in-differences (DID) analysis to compare the MCH interventions between Kenge and Boko from baseline to endline. The binary logistic DID model included dependent, independent, and control variables.

The dependent variable in the binary logistic DID analysis was the 4^+^ ANC services utilization. To measure this dependent variable, women were asked: *“How many times did you visit a health facility for ANC before your last delivery?”* A response of “four times or more than four times” was coded “1”; others were coded “0.”

The independent variables in binary logistic DID analysis used a dummy variable including time, group, and an interaction variable that multiplied the time variable by the group variable. To improve the accuracy of the analysis, we controlled for socio-demographic variables including the number of household members, the number of children under the age of five, mother’s reading skills, writing skills, health insurance, and monthly household income. We used reading and writing skills rather than education level because we installed informational signboards on how to visit the health facilities, and wanted to focus on the effects of the promotional campaign. Therefore, the effects of ANC services by interventions were δ in Table 2 [20–23].

**Table 2:** Intervention effects of the binary logistic regression DID analysis

Group	Time	
	Baseline (2014)	Endline (2017)
Intervention (①)	α + γ + Χ + ε	α + γ + β + δ + Χ + ε
Control(②)	α + Χ + ε	α + β + Χ + ε
Difference(①-②)	γ	γ + δ
Intervention effect		δ

## Results

### Characteristics of study participants

A total of 2279 reproductive-aged women between the intervention and control group participated. Thus, out of 2279 women respondents, data of 2085 women (91.4%) were analyzed. As depicted in Table 3, the mean age of respondents in Kenge was 28.7 years in the baseline, 29.6 years in the endline. Women in Boko had the similar mean age. Some 86.8% of the women in Kenge and 81.8% of women in Boko had higher than or equal to secondary levels of education in the baseline. And 82.7% of women in Kenge and 73.1% women in Boko had higher than or equal to secondary levels of education in the endline. Thus, more than 80% of the respondents in Kenge and Boko completed higher than secondary level education. Some 41.4 and 53.4% of the women in Kenge had received 4+ ANC services in the baseline and endline, respectively. In contrast, 31.3 and 43.4% women in Boko had received these services in the baseline and endline, respectively.

**Table 3:** Characteristics of 2085 reproductive-aged women respondents in the baseline (2014) and in the endline (2017) across the intervention and control sites in the Democratic Republic of Congo

	Baseline (2014)		Endline (2017)	
	Intervention	Control	Intervention	Control
Mean ± SD				
Respondent age	28.7 ± 6.7	28.6 ± 7.2	29.6 ± 6.9	29.4 ± 7.1
Childbirth	3.4 ± 2.1	3.4 ± 2.2	3.9 ± 2.4	3.9 ± 2.4
No. of household members	5.6 ± 2.2	5.2 ± 2.1	6.0 ± 2.3	5.8 ± 2.2
No. of children under 5	1.8 ± 0.8	1.8 ± 0.7	1.7 ± 0.7	1.8 ± 0.9
Monthly income ($)	64.7 ± 104.9	52.9 ± 83.9	51.3 ± 66.9	45.0 ± 63.6
Monthly expenditures ($)	16.7 ± 25.8	23.5 ± 35.0	12.7 ± 19.8	13.2 ± 37.0
Quality of life	67.2 ± 13.7	64.3 ± 12.6	68.7 ± 15.8	66.1 ± 15.0
Frequency (%)				
Antenatal care (≥4 times)	229 (41.4%)	41 (31.3%)	370 (53.4%)	251 (43.4%)
Educational level (≥ secondary)	425 (86.8%)	81 (81.8%)	594 (82.7%)	449 (73.1%)
Reading skills	349 (63.7%)	64 (49.6%)	434 (63.8%)	275 (49.8%)
Writing skills	349 (63.7%)	62 (48.1%)	424 (62.4%)	269 (48.7%)
Health insurance	31 (6.8%)	4 (2.7%)	19 (2.7%)	16 (2.7%)
No. of women respondents	602	150	719	614

### Binary logistic DID analysis

In order to identify the effect of the MCH interventions on the use of 4^+^ ANC services, this study performed a binary logistic regression DID analysis between Kenge and Boko (Table 4). The odds of reproductive-aged women’s 4+ ANC service utilization at the intervention site in the endline increased 2.054 times from the baseline (OR: 2.054, 95% CI: 1.365–3.092, *p* < .001) as compared to the control site in Model 1, which only included Time, Group, Time × Group (δ). Model 2 used the same independent variables and also controlled for additional variables including the number of household members, number of children under five, reading skills, writing skills, insurance, and income. After accounting for these controlled variables in Model 2, the odds of reproductive-aged women’s 4+ ANC service utilization at the intervention site in the endline increased 2.280 times from the baseline (OR: 2.280, 95% CI: 1.332–3.902, *p* = .003) as compared to that in the control site.

**Table 4:** Binary logistic regression difference-in-differences analysis of 4^+^ ANC services. (Baseline N: 752; Endline N: 1333; Total N: 2085)

Variable		Model 1					Model 2				
		B	S.E.	ORs	95% CI	P	B	S.E.	ORs	95% CI	P
Time (β)	Baseline	Ref.					Ref.				
	Endline	−.362	.239	.696	[.430–1.112]	.129	.212	.298	1.237	[.690–2.217]	.476
Group	Control	Ref.					Ref.				
	Intervention	.453	.209	1.573	[1.044–2.371]	.030	.478	.260	1.612	[.969–2.683]	.066
Time × Group (*δ*)		.720	.209	2.054	[1.365–3.092]	.001	.824	.274	2.280	[1.332–3.902]	.003
No. of household members							−.008	.028	.992	[.940–1.048]	.786
No. of children under 5							.005	.078	.1005	[.863–1.171]	.004
Reading skills	NO						Ref.				
	YES						.728	.548	2.072	[.707–6.068]	.184
Writing skills	NO						Ref.				
	YES						−.743	.545	.476	[.163–1.385]	.173
Insurance	NO						Ref.				
	YES						−.895	.306	.408	[.224–.744]	.003
Income (US $)							.000	.001	1.000	[.999–1.002]	.745
Constant		−.800	.190	.449			.035	.394	1.036		

## Discussion

This study aimed to identify the MCH intervention effect on 4^+^ ANC service utilization among reproductive-aged women in the DRC. The use of 4^+^ ANC services increased 2.280 times as a result of the intervention.

The characteristics of this study, childbirth in the life of reproductive-aged women in Kenge were identified on average 6.0 times that is similar to the 6.0 times reported by the World Bank in 2015 [24]. The education levels of respondents showed that more than 70% of reproductive-aged women in Kenge and Boko had completed secondary or higher levels of education. On the other hand, only 48% of reproductive-aged women in the completed higher than secondary levels of education [25].. This result shows that reproductive-aged women in Kenge and Boko have higher education levels than women living in other cities across the DRC. A previous study showed that interventions are more effective for reproductive-aged women with higher education levels [26]. Therefore, the interventions activities might have been effective due to women’s higher levels of education at Kenge. The monthly household income in Boko was lower than in Kenge, in part due to the UN implementing income generating activities in Kenge in 2014, which might have increased their monthly household income [27].

The results of this study indicated that the average medical expenditure in Kenge was $152.4 per year, and the medical expenditure in Boko was $158.4. The medical expenditures in Kenge and Boko were higher than the average ($128) in rural areas in the DRC but were lower than medical expenditure in urban areas ($376) and the overall average in the DRC ($206) [25].

This study provided interventions in Kenge based on the three delays model of factors that impede women from seeking the MCH, increasing the MMR. The first delay involves the delay in making decision to seek care. This delay stems from lack of awareness of services and health facility, which may be addressed by improving the educational interventions focused on decision-making behaviors. Therefore, we provided education programs to change the behavior of reproductive-aged women. Previous studies have shown that the fastest and most effective MCH interventions are those that focus on behavioral changes among reproductive-aged women [28]. Accordingly, a qualitative study in Kenge confirmed that the radio education intervention in the first phase was effective [29].

The second phase of delay involves the delay in identifying and ultimately reaching the medical facilities. According to a previous study in Tanzania, reproductive-aged women know that ANC is very important, but pregnant women find it difficult to visit the health facility at night because of dangerous animals and inequity of access to health facilities. Additionally, health workers interviewed in a previous study stated that “… I would like them to attend ANC from at least 8 weeks [of pregnancy], if we have a chance to identify the pregnancy that early then I think this lady has a chance to receive a good health service” [30]. In order to enhance access to health facilities, we provided emergency transportation, including ambulances and motorcycles, and these interventions were effective at increasing the number of health facility visit. However, unlike other studies [30] we did not calculate the accessibility cost of transportation; future studies need to calculate this cost to enable evidence-based decision-making.

The third phase of delay involves the delay in receiving adequate and appropriate treatment. In previous studies, the mortality of the mother or the children occurred in the third phase in the three delays model [30, 31]. Results from this study indicated that MCH interventions had an effect of increasing 4^+^ ANC services utilization by 2.28 times. Based on previous studies, reproductive-aged women who received 4+ ANC have a lower mortality rate than reproductive-aged women with 4^−^ ANC. Moreover, children under five who were born from a mother that received 4+ ANC services have a lower mortality rate [32, 33]. Therefore, it is expected that newborns that are born after this intervention will have positive effects on their health. Future studies in Kenge must conduct a study on the intervention’s effect on newborns .

Some issues may have affected the effectiveness of the interventions from 2014 to 2017. According to an announcement by WHO, there was a measles outbreak in the DRC in 2015. The outbreak occurred in the province of Katanga, which spread rapidly to nearby Kenge and Boko and resulted in 5000 deaths and 300,000 measles patients [34]. We assumed that this could be the cause of the decrease in the intervention effect in the second year. Because health facilities in Kenge are widely known to provide medical interventions, patients from other areas overcrowded these facilities, thus, pregnant women at the intervention site might not have received adequate ANC services.

According to previous studies, it was found that the main issue with MCH interventions in low-income countries is the frequent turnover of health workers [28]. Doctors, chief nurses, pharmacists, and midwives in Kenge had turnover around every 2 years. We confirmed that some of the health workers who had completed their intervention training had been moved out to other areas, which may have reduced specific possible outcomes in the intervention area due to provider turnover.

Many low-income countries, international organizations, and NGOs have been implementing the official development assistance (ODA) projects in the DRC. There are various focuses of ODA projects, such as health, construction, agriculture, education, and others. Despite the efforts of the ODA projects by international organizations and NGOs, the number of projects has been reduced due to safety issues in the DRC, such as civil war. And the DRC still lacks evidence studies on public health issues such as MCH, Ebola, and malaria. Although this study was conducted in one region of the DRC, it is expected to provide evidence for low-income countries, international organizations, NGOs, and research institutions to implement the MCH project across the DRC.

### Strengths and limitations of the study

This study focused on MCH interventions based on the three delays model for reproductive-aged women in the DRC. The three delays model targets interventions to reduce the MMR by identifying the three phases that affect MCH. However, this study used the number of ANC services as dependent variable, not the MMR. For this reason, it was difficult to obtain the MMR data from the health facility, survey, and public data in the DRC, and we doubted the reliability of the obtained MMR data. Future studies must use the MMR indicator to identify the MCH intervention effect.

According to previous research, it is necessary to select the control area for conducting the DID analysis, which has similar characteristics to the intervention area before the study is implemented [35]. To test the homogeneity of the two areas, previous studies have used the Propensity Score Matching (PSM) method or Inverse Probability of Treatment Weighting (IPTW) [36, 37]. However, this study used the chi-squared test of homogeneity because the PSM method would have decreased the sample size. In future studies, PSM method should be considered while calculating the sample size. In the baseline survey, 312 households in Boko were the control group from 10 AS, but only 150 households were analyzed because of missing data. Therefore, the sample size for analysis was not balanced. Future research should get balanced sampling by using RUSBoost or over-sampling methods [38].

In a previous study, the evaluation of intervention activities were conducted using multi-level analysis, suggesting standardized indicators [28]. This study, however, was conducted at a single level. Therefore, multi-level, DID analyses might be conducted to account for higher-level factors in the future.

The results show that the MCH project using the three delays model as framework was able to bring an improvement to reproductive-aged women’s 4+ ANC service utilization. ANC is an effective intervention for reducing MMR, so we expected this study to also reduce the MMR. In the future, we expect these results to inform the MoPH MCH policies in the DRC.

## Conclusion

This study examined the effect of the MCH intervention based on the three delays model from 2014 to 2017 in Kenge, DRC. According to the WHO and the MoPH, less than half of reproductive-aged women in the DRC received 4+ ANC services [5]. According to previous studies, ANC is an effective intervention for reducing MMR [9]. As a result of implementing MCH intervention activities using the three delays model, the number of 4+ ANC visits increased 2.280 times from the baseline. Currently, there is a lack of research using the three delays model. Women who received 4+ ANC services had a lower mortality, so this study was expected to also show a reduced MMR. Therefore, we expect the findings of this study to inform the MCH policies by the MoPH in the DRC

## Data Availability

The data used for the current study is available from the corresponding author on reasonable request.

## Abbreviations

4^+^ ANC: More than four visits to antenatal care services
AS: Aires de santé
CHAID: Chi-squared Automatic Interaction Detection
DID: Difference-in-differences
IPTW: Inverse Probability of Treatment Weighting
MCH: Maternal and Child Healthcare
MMR: Maternal Mortality Ratio
MNU: Maternal and Newborn care Unit
PNC: Postnatal care
PSM: Propensity Score Matching
RECO: Relais Communautaires
SDGs: Sustainable Developments Goals
